# Cost-effectiveness of nutritional intervention in elderly subjects after hip fracture. A randomized controlled trial

**DOI:** 10.1007/s00198-012-2009-7

**Published:** 2012-05-26

**Authors:** C. E. Wyers, P. L. M. Reijven, S. M. A. A. Evers, P. C. Willems, I. C. Heyligers, A. D. Verburg, S. van Helden, P. C. Dagnelie

**Affiliations:** 1 Department of Epidemiology, CAPHRI School for Public Health and Primary Care, Maastricht University Medical Centre, P.O. Box 616, 6200 MD Maastricht, The Netherlands; 2 Department of Dietetics, Maastricht University Medical Centre, P.O. Box 5800, 6202 AZ Maastricht, The Netherlands; 3 Department of Health Services Research, CAPHRI School for Public Health and Primary Care, Maastricht University Medical Centre, P.O. Box 616, 6200 MD Maastricht, The Netherlands; 4 Department of Orthopedic Surgery, CAPHRI School for Public Health and Primary Care, Maastricht University Medical Centre, P.O. Box 5800, 6202 AZ Maastricht, The Netherlands; 5 Department of Orthopedic Surgery, Atrium Medical Centre, P.O. Box 4446, 6401 CX Heerlen, The Netherlands; 6 Department of Orthopedic Surgery, Orbis Medical Centre, P.O. Box 5500, 6130 MB Sittard, The Netherlands; 7 Department of Trauma Surgery, Maastricht University Medical Centre, P.O. Box 5800, 6202 AZ Maastricht, The Netherlands; 8 Present Address: Department of Trauma surgery, Isala Klinieken, P.O. Box 10400, 8000 GK Zwolle, The Netherlands

**Keywords:** Cost-effectiveness, Elderly, Hip fracture, Nutritional support, Quality of life

## Abstract

**Summary:**

Hip fracture patients can benefit from nutritional supplementation during their recovery. Up to now, cost-effectiveness evaluation of nutritional intervention in these patients has not been performed. Costs of nutritional intervention are relatively low as compared with medical costs. Cost-effectiveness evaluation shows that nutritional intervention is likely to be cost-effective.

**Introduction:**

Previous research on the effect of nutritional intervention on clinical outcome in hip fracture patients yielded contradictory results. Cost-effectiveness of nutritional intervention in these patients remains unknown. The aim of this study was to evaluate cost-effectiveness of nutritional intervention in elderly subjects after hip fracture from a societal perspective.

**Methods:**

Open-label, multi-centre randomized controlled trial investigating cost-effectiveness of intensive nutritional intervention comprising regular dietetic counseling and oral nutritional supplementation for 3 months postoperatively. Patients allocated to the control group received care as usual. Costs, weight and quality of life were measured at baseline and at 3 and 6 months postoperatively. Incremental cost-effectiveness ratios (ICERs) were calculated for weight at 3 months and quality adjusted life years (QALYs) at 6 months postoperatively.

**Results:**

Of 152 patients enrolled, 73 were randomized to the intervention group and 79 to the control group. Mean costs of the nutritional intervention was 613 Euro. Total costs and subcategories of costs were not significantly different between both groups. Based on bootstrapping of ICERs, the nutritional intervention was likely to be cost-effective for weight as outcome over the 3-month intervention period, regardless of nutritional status at baseline. With QALYs as outcome, the probability for the nutritional intervention being cost-effective was relatively low, except in subjects aged below 75 years.

**Conclusion:**

Intensive nutritional intervention in elderly hip fracture patients is likely to be cost-effective for weight but not for QALYs. Future cost-effectiveness studies should incorporate outcome measures appropriate for elderly patients, such as functional limitations and other relevant outcome parameters for elderly.

## Introduction

In The Netherlands, as well as in other countries, the incidence of hip fractures in the elderly is high, and it is expected to increase in the nearby future. Hip fractures are one of the most common reasons for hospital admission and transfers to nursing facilities in the elderly [1]. After hip fracture, only 37% of the patients will return to their pre-fracture functional status leading to high health care costs and a major burden on health care utilization [2]. Not only costs resulting from a hip fracture during hospital stay are relevant, but also long-term costs such as recovery in a rehabilitation clinic, the need for home care, and the increased burden on informal care givers which may play an even more important role [2, 3].

At the time of hospital admission for surgical treatment of their hip fracture, hip fracture patients are reported to be malnourished, and the nutritional status can deteriorate further during hospital admission because of a spontaneous reduction in food intake due to lack of appetite or nausea [4–9]. Malnutrition in hip fracture patients is reported to be associated with impaired muscle function, disability, loss of independency, decreased quality of life, delayed wound healing, higher complication rate, prolonged rehabilitation time, and increased mortality rate [7, 8, 10–17]. Both hip fracture patients and malnourished patients in general have an increased use of health care as compared with well-nourished and non-fracture patients, and it is expected that it would result in higher health care costs [18–21]. Early treatment of malnutrition is of vital importance to minimize losses and to achieve rapid weight recovery after hip fracture.

In the past decades, several studies have been conducted to determine the effectiveness of nutritional supplementation on length of stay, postoperative complications, mortality, nutritional status and functional status. Furthermore, within the past decades, economic evaluations have gained more and more attention, and their importance has increased because of the continuous rising health care expenses and the limited budgets available. As a consequence, new or additional treatments should not only have to be effective but also cost-effective. Previous research on costs and cost-effectiveness of nutritional support or intervention is scarce. A few studies have shown that health care costs can be reduced by nutritional support in malnourished elderly [20, 22, 23]. Kruizenga et al. [24] reported that nutritional screening and treatment of malnourished patients at an early stage of hospitalization is cost-effective.

Although several studies have shown the effectiveness of nutritional support in elderly hip fracture patients, none of these studies have incorporated an economic or cost-effectiveness evaluation. Therefore, the aim of the present study was to investigate the cost-effectiveness of an intensive dietary intervention comprising combined dietetic counseling and oral nutritional supplementation, as compared with usual nutritional care in elderly subjects after hip fracture from a societal perspective with a time horizon of 6 months.

## Methods

### Subjects

Eligible were patients admitted for surgical treatment of hip fracture, aged ≥55 years [25]. Patients were excluded if they had a pathological or periprosthetic fracture; a disease of bone metabolism (e.g. M Paget, M Kahler, hyperparathyroidism); an estimated life expectancy <1 year due to underlying disease; if they used an oral nutritional supplement before hospital admission; if they were unable to speak Dutch, lived outside the region or had been bedridden before their hip fracture. Patients were also excluded if they had dementia or were cognitively impaired, defined as a score of <7 on the Abbreviated Mental Test, as assessed before inclusion [26].

### Design

The present economic evaluation was embedded in an open-label parallel multi-centre, randomized controlled trial on the effectiveness of nutritional intervention in elderly subjects after a hip fracture [25]. The economic evaluation was performed from a societal perspective using a time horizon of 6 months.

For patient recruitment, we made a daily inventory of all hip fracture patients admitted to the surgical and orthopedic wards of Maastricht University Medical Centre (Maastricht), Atrium Medical Centre (Heerlen) and Orbis Medical Centre (Sittard). Eligible patients who met the inclusion criteria were invited to participate, and written informed consent was obtained within 5 days after surgery. After informed consent and baseline measurements, patients were randomized according to a concealed computer-generated random-number sequence list after pre-stratification for hospital, gender and age (55–74 vs. ≥75 years) with an allocation ratio of 1:1. After randomization, all patients were visited by a study dietician who evaluated patients’ nutritional intake by a 24-h recall. Then, patients allocated to the intervention group received dietetic counseling and an oral nutritional supplement as needed, for 3 months after fracture, whereas patients in the control group received usual nutritional care. Costs and outcome measurements were assessed at 3 and 6 months postoperatively [25]. Patients were discharged from the hospital according to standard care, either to a rehabilitation clinic or to the patient’s home with home care, or to the nursing home or elderly home where they had lived there before hospitalization. The study was approved by the Medical Ethical Committee of Maastricht University Hospital and Maastricht University and conducted according to the Declaration of Helsinki.

### Nutritional intervention

Patients in the intervention group received a combination of frequent dietetic counseling and consumption of a multi-nutrient oral nutritional supplement (ONS), starting during hospital admission and continued in the rehabilitation centre and/or at home, until 3 months after hip fracture surgery.

A dietician visited each patient twice during their hospital stay. At the first visit, the dietician took a 24-h recall of the patient’s diet during hospitalization. To optimize normal food intake, all patients received an energy- and protein-enriched diet, and recommendations were given with regard to choice, quantity and timing of food products. In addition, patients were advised to consume two bottles of ONS daily in-between the main meals. The ONS was a milk-protein based, or a yoghurt- or juice-style supplement (Cubitan, Nutridrink Yoghurt style, or Nutridrink Juice style, N.V. Nutricia, Zoetermeer, The Netherlands) providing 2.1 MJ (500 kcal) and 40 g of protein per 500 ml. Furthermore, the dietician made arrangements to solve any problems, e.g. feeding difficulties, in collaboration with the hospital medical and nursing staff.

At the second visit during hospitalization, 7–8 days after surgery, the dietician evaluated food intake and the consumption of the ONS using a 24-h recall and gave individually tailored advice to optimize dietary intake. Furthermore, the transfer of the patient to the rehabilitation centre or the patient’s home was prepared by evaluating the patient’s physical restrictions with regard to nutritional care, i.e. purchasing food products and the preparation of meals, and by making arrangements to enable adequate food intake, e.g. support of informal caregivers and delivery of information on meal services.

After hospital discharge, the dietician visited each patient three times (1, 2 and 6 weeks after discharge) at the patient’s home or in the rehabilitation centre (whatever was applicable) in order to evaluate dietary intake including the intake of the ONS, to evaluate possible bottlenecks in nutritional care at home (e.g. shopping, cooking) and to give dietary advice as needed. In addition, in-between these home visits, weekly telephone calls were made (3, 4, 5, 8 and 10 weeks after discharge) to evaluate dietary intake (including the ONS) by 24-h recall. If necessary, a telephone call was replaced by a home visit.

### Usual care

Patients allocated to the control group received usual care as provided in the hospital, rehabilitation clinic or at home, i.e. dietetic care or nutritional supplements were only provided on demand of the medical doctor in charge. In the control group, ten patients (13%) received ONS and 18 patients (23%) received dietetic counseling.

### Economic evaluation

#### Effect measures

##### Weight

At baseline, self-reported weight was used, because patients were not able to stand on a weighing scale because of hip fracture. At 3 months postoperatively, weight was measured using an electronic weighing scale (Seca 862, Seca Ltd, Birmingham, UK). The difference in weight in kilograms between baseline and 3 months postoperatively was calculated and used to evaluate the effectiveness of the nutritional intervention.

##### Quality adjusted life years

Quality of Life was estimated at baseline and at 3 and 6 months postoperatively using the Dutch version of EuroQoL (EQ-5D-3 L) [27–29]. In the EuroQoL, the patient was asked to make a statement on the degree of problems (no problem, some problems or major problems) he/she experienced on the dimensions of mobility, self-care, usual activities, pain or discomfort and anxiety or depression. The degree of problems on each dimension were combined to a health state. Based on these health states, utilities were calculated based on the social tariff by Dolan because this is the internationally accepted standard [30]. Utilities are the preferences that individuals or the society may have for a particular set of health outcomes. These utilities were used to calculate Quality Adjusted Life Years (QALYs), which are defined as ‘a measure of a person’s length of life weighted by a valuation of their health related quality of life’ [31]. QALYs are used to make a comparison between the effects of different treatments and to evaluate cost-effectiveness of interventions. The value of the QALY can range from below zero, representing the worst possible health state, up to 1, representing the best possible health state.

#### Cost measures

Medical and non-medical costs were measured at baseline and at 3 and 6 months postoperatively using a standardized 3-month retrospective patient costing questionnaire. Patients were asked to report the frequency and location of consultation with the general practitioner, physiotherapist and other paramedical care givers, as well as professional homecare for assistance with activities of daily living and household activities of daily living, and assistant devices and medical aids. Medication was registered from the patient’s medical chart, the medication list as provided by the general practitioner or pharmacy, supplemented by registration of medication packages. Length of stay in hospital, rehabilitation clinic, nursing home and home for the elderly were calculated using admission and discharge dates. The number and duration of face-to-face visits and telephone calls were calculated using the dietician’s time registries and used to calculate the costs of a face-to-face visit and telephone call. The quantity of the ONS was calculated based on the number of ONS as advised by the dietician.

We assessed nutritional intervention costs, health-care-related costs and patient and family costs. Nutritional intervention costs were defined as the costs of the dietetic counseling by the dietician (face-to-face visits and telephone calls) and nutritional supplementation (oral nutritional supplements and tube feeding). Health-care-related costs were hospital-related costs (hospital admissions and outpatient specialist care), other in-patient-related costs (admissions to rehabilitation clinic, nursing home or home for the elderly and day centre activities), general practitioners, paramedical care (physiotherapy, occupational therapy, other alternative therapies), professional home care, assistant devices and medical aids and prescribed and over-the-counter medication. Patient and family costs included the costs of home adjustments, paid domestic help and meal services. Productivity costs were considered irrelevant for this population because 89% of the patients in the control group and 96% of the patients in the intervention group were retired; therefore, these costs were not included in the calculation.

To calculate the costs, the volumes of each cost category were multiplied by the cost price of each cost category. Cost prices, presented in Euros, were based on the “Dutch manual for costing: methods and standard costs for economic evaluations in healthcare” for the year 2010 [32]. Standardized cost prices were used where available, or else real costs or tariffs were used to estimate the costs. Medication costs were calculated using prices based on the Defined Daily Dose which is defined by the Health Care Insurance Board as the assumed average maintenance dose per day for a drug used for its main indication in adults [33, 34]. Prices of paid domestic help were based on tariffs for unpaid work. With respect to costs of hospital admissions, the cost price of a non-teaching hospital was used because hip fracture surgery does not require the expertise of a teaching hospital, and the Maastricht University Medical Centre has both the function of a non-teaching and teaching hospital. Costs of surgery were not included in the cost calculation because previous research by Haentjens et al. [35] showed that the costs of the different types of surgery are comparable.

#### Incremental cost-effectiveness ratios, cost-effectiveness planes and cost-effectiveness acceptability curves

To evaluate cost-effectiveness, incremental cost-effectiveness ratios (ICERs) were calculated. ICERs were calculated by dividing the difference in the mean costs (between two treatments or interventions) by the differences in the mean outcomes. In this study, ICERs were calculated for weight change and for QALYs. The ICERs were interpreted as the incremental cost per unit of additional outcome [29, 36].

These ICERs were plotted in a cost-effectiveness plane (CEP), in which the *x*-axis showed the difference in effect between the interventions and the *y*-axis the differences in costs between the interventions [29, 36, 37]. In the CEP, four quadrants were shown; ICERs located in the North East (NE) indicated that the intervention was more effective and more costly as compared with usual care. ICERs in the South East (SE), the dominant quadrant, indicated that the intervention is more effective and less costly. ICERs in the South West (SW) indicated that the intervention was less effective and less costly, and ICERs located in the North West (NW) indicated that the nutritional intervention was less effective but more costly.

Based on the CEPs, cost-effectiveness acceptability curves (CEAC) were plotted [29, 36–38]. In the CEAC, the probability that the nutritional intervention is more cost-effective as compared with the usual care (*y*-axis) was presented for several ceiling ratios (*x*-axis), which were defined as the amount of money the society is willing to pay to gain one unit of effect [29, 36–38]. Within The Netherlands, the value the society is willing to pay to gain one QALY ranges from 20,000 to 80,000 Euro, depending on the severity of the disease [39].

#### Sensitivity analyses

Sensitivity analyses were performed for age categories (55–74 vs. ≥75 years) because elderly patients can have more co-morbidities and postoperative complications as compared with younger patients.

Sensitivity analyses were also performed for patients classified according to their risk of malnutrition at baseline, as measured by the Mini Nutritional Assessment (MNA). The MNA was developed for elderly people and includes 18 items grouped in four categories: anthropometric assessment (including BMI, weight loss, arm circumference and calf circumference); general assessment of lifestyle, medication use, mobility, presence of signs of depression or dementia); short dietary assessment (number of meals, food and fluid intake, autonomy of feeding) and subjective assessment (self perception of health and nutrition) [40, 41]. A score of ≥24 indicates no malnutrition; a score between 17 and 23.5 indicates being at risk of malnutrition, and a score less than 17 indicates malnutrition. For this purpose, the group malnutrition and the group at risk of malnutrition are combined and compared with the group no malnutrition.

#### Statistical analysis

Data were analyzed using SPSS version 15 and Excel 2003 and based on the intention-to-treat principle. Missing values for the EuroQoL at 6 months postoperatively were imputed by last observation carried forward. If volume date were missing to calculate the costs, these missing data were replaced by individual means of valid volume data before multiplying the volumes by the cost prices. Costs were presented as means and standard deviations, and Mann–Whitney *U* tests were used to test for significant differences in costs between the intervention and control group. The robustness of the cost analyses was also tested by bootstrapping (1,000×). Furthermore, bootstrapping (5,000×) was used to calculate the uncertainty around the cost-effectiveness ratios, and CEPs and CEACs were plotted [29, 36–38]. Sensitivity analyses were performed for age categories (55–74 vs. ≥75 years) and for the risk of malnutrition at baseline (at risk of malnutrition and malnutrition vs. no malnutrition). Bootstrapping was also used to calculate the uncertainty around the ICERs resulting from the sensitivity analyses, and CEPs and CEACs were also plotted.

## Results

From July 2007 until December 2009, a total of 1,304 hip fracture patients were admitted to the surgical and orthopedic wards of the participating hospitals and screened for eligibility. Of the screened patients, 895 (69%) did not meet the inclusion criteria, mainly due to cognitive impairment (52%). Two-hundred fifty-seven (20%) patients refused to participate. Of the resulting 152 patients who gave informed consent, 73 were randomly allocated to the intervention group and 79 to the control group. During the 3-month intervention period, seven patients (four, intervention; three, control) passed away, and seven patients (three, intervention; four, control) withdrew their participation, resulting in 138 assessable patients (68 intervention, 72 control) at 3 months. During the follow-up (3–6 months after surgery), four patients (two intervention, two control) passed away, and three patients (one, intervention; two, control) withdrew their participation, resulting in 63 patients in the intervention group and 68 patients in the control group who completed follow-up.

At baseline, the intervention and control group were comparable with respect to gender and age. In both groups, the majority of the patients sustained a fracture of the medial neck of the femur. In the intervention group, more patients had received gamma nail, and fewer patients had received hemi-arthroplasty as compared with the control group (Table 1). After hospitalization, in the intervention group as well as in the control group, 42 patients were discharged to a rehabilitation clinic. At baseline, 37% of the patients in the intervention group were malnourished or at risk of malnutrition as compared with 48% of the patients in the control group. Medical costs measured at baseline over a 3-month period, before hip fracture, were comparable between both groups (data not shown).

**Table 1:** Baseline characteristics

	Intervention group		Control group	
	(*n* = 73)		(*n* = 79)	
	*n*	(%)	*n*	(%)
Sex				
Female	54	(74)	54	(68)
Male	19	(26)	25	(32)
Age	79	(55–93)	78	(57–94)
Type of residence before fracture				
Home	63	(86)	66	(83)
Nursing home	2	(3)	4	(5)
Home for the elderly	8	(11)	7	(9)
Rehabilitation clinic/hospital	0	(0)	2	(3)
Fracture type				
Medial neck	36	(49)	45	(57)
Pertrochanteric	32	(44)	33	(42)
Subtrochanteric	5	(7)	1	(1)
Type of surgery				
Gamma nail	37	(51)	24	(30)
Dynamic hip screw	6	(8)	11	(14)
Hemiarthroplasty	19	(26)	30	(38)
Total hip replacement	4	(5)	7	(9)
Three cannulated screws	7	(10)	6	(8)
Femoral nail	0	(0)	1	(1)
MNA^a^				
No malnutrition	46	(63)	41	(52)
At risk of malnutrition or malnourished	27	(37)	38	(48)

^a^Mini Nutritional Assessment

### Costs

As shown in Table 2, the mean cost of the nutritional intervention per patient in the intervention group was 613 Euro. Several patients in the control group also received dietetic counseling and ONS, with mean cost of 88 Euro (*p* = 0.000). The additional costs of the nutritional intervention were only 3% of the total costs and were thus relatively low as compared with other health-care-related costs and patient- and family-related costs. Total health care costs, patient and family costs, as well as the subcategories of these costs, were not significantly different between both groups.

**Table 2:** Mean costs in Euro

Cost category	Intervention group (*n* = 73)					Control group (*n* = 79)					*t* test	Bootstrap 95% Uncertainty interval	
	Mean	SD	Median	Min^a^	Max^b^	Mean	SD	Median	Min^a^	Max^b^	*p* value	2.5th percentile	97.5th percentile
Nutritional intervention	613	258	586	30	1,352	88	311	0	0	2,187	0.000	433	608
Dietetic counseling	244	55	243	30	374	22	50	0	0	269	0.000	206	237
Oral nutritional supplement	370	225	346	0	1,095	67	269	0	0	1,918	0.000	219	381
Health-care-related	22,449	16,003	20,577	2,911	73,719	22,491	16,741	21,470	2,332	73,362	0.849	−5,203	5,119
Hospital-related	7,072	5,112	5,482	1,892	25,001	6,492	6,255	4,977	1,516	51,129	0.342	−1,282	2.85
Other inpatient-related	10,967	12,783	10,677	0	59,929	11,481	14,032	8,266	0	57,863	0.959	−4,677	3,468
General practitioner	131	190	71	0	1,045	118	164	85	0	1,089	0.900	−43	71
Paramedical care	1,692	1,240	1,741	0	6,219	1,761	1,379	1,700	0	7,421	0.962	−493	362
Professional home care	1,743	2,465	156	0	10,187	1,660	2,519	0	0	9,919	0.718	−600	865
Assistive devices and medical aids	531	1,393	103	0	8,466	662	1,395	193	0	5,383	0.843	−719	823
Medication	314	391	182	0	1,923	316	384	175	0	1,897	0.943	−120	125
Patient- and family-related	291	568	0	0	3,216	317	585	0	0	3,267	0.959	−208	158
Home adjustments	54	264	0	0	1,545	53	262	0	0	2,162	0.450	−87	89
Paid domestic help	161	393	0	0	1,823	185	491	0	0	3,267	0.782	165	115
Meal services	76	207	0	0	927	79	218	0	0	930	0.868	−201	175
Total	23,353	16,124	21,446	3,497	74,054	22,896	16,834	21,470	2,332	73,362	0.665	−4,604	5,827

^a^Minimum

^b^Maximum

### Cost-effectiveness

#### Weight as outcome

**Fig. 1:**
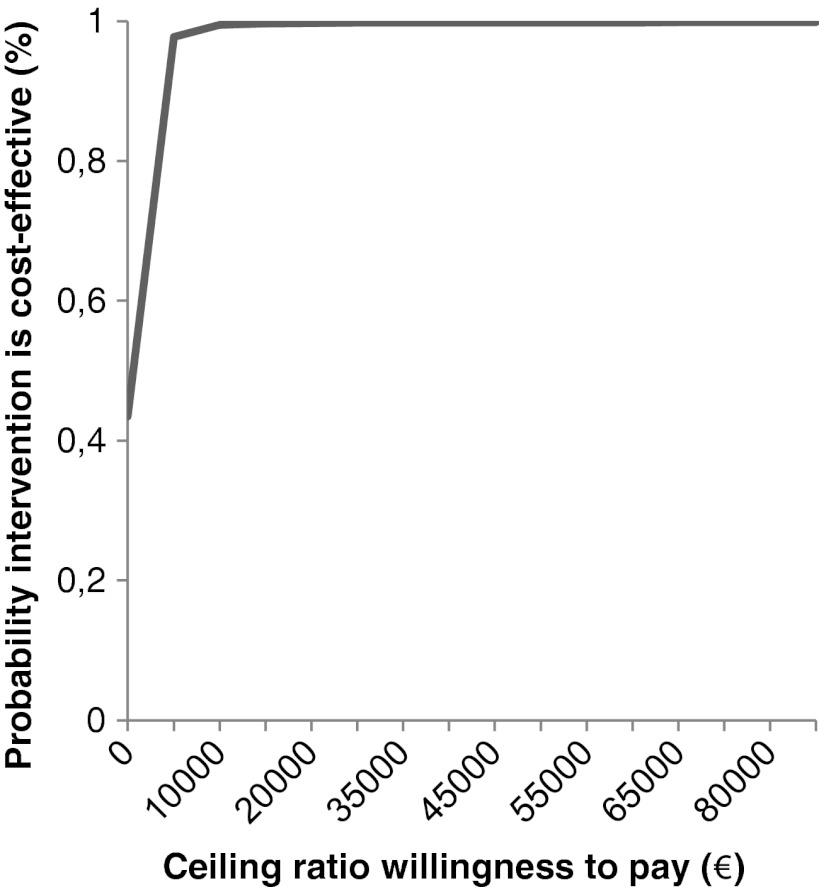
Cost-effectiveness acceptability curve presenting the probability that the nutritional intervention is cost-effective (*y*-axis) for weight increase, given various ceiling ratios for willingness to pay (*x*-axis)

The intervention effect for weight, defined as the difference in change between the intervention and control group from baseline to 3 months postoperatively has a statistically significant positive value, meaning that the patients in the intervention group gained more weight as compared with patients in the control group. The estimated intervention effect from baseline to 3 months postoperatively was 1.91 kg (95% CI, 0.60–3.22; *p* = 0.005). The ICER for total societal costs per kilogram weight increase was 241 Euro. As presented in Table 3, the overwhelming majority of the dots in the CEP were located in the NE and SE quadrant. The ICERs located in the NE quadrant represent ratios indicating that the nutritional intervention was more costly and more effective as compared with usual care. The ICERs located in the SE represent ratios indicating that the nutritional intervention was less costly and more effective as compared with usual care. The CEAC (Fig. 1) indicates that, with a willingness to pay of 5,000 Euro, the probability that the nutritional intervention was cost-effective based on its total societal costs per kilogram weight was as high as 98%. Even at a willingness to pay € 2,500, the intervention was still ∼70% likely to be cost-effective.

**Table 3:** Cost-effectiveness analyses for weight difference and QALY

	Participants			Distribution on cost-effectiveness plane			
	Intervention	Control	ICER^a^	NE^b^%	SE^c^%	SW^d^%	NW^e^%
Weight							
Weight base case	65	72	241	56	43	0	0
55–74 years	22	27	−2,788	27	17	27	29
75 years and above	43	45	149	56	44	0	0
No malnutrition^f^	42	38	2,349	93	7	0	0
(At risk of) malnourished^f^	23	34	−1,404	18	82	0	0
QALY^g^							
QALY base case	62	69	36,943	42	31	5	22
55–74 years	20	28	−4,880	40	60	0	0
75 years and above	42	41	−104,521	22	13	12	52
No malnutrition^f^	40	39	60,300	74	14	0	12
(At risk of) malnourished^f^	22	30	−67,577	14	10	12	64

^a^Incremental cost-effectiveness ratio

^b^North East quadrant: the intervention was more effective and more costly as compared to usual care

^c^South East quadrant: the intervention was more effective and less costly as compared to usual care

^e^North West quadrant: the intervention was less effective and more costly as compared to usual care

^d^South West quadrant: the intervention was less effective and less costly as compared to usual care

^f^According to Mini Nutritional Assessment (MNA)

^g^Quality adjusted life years

#### QALYs as outcome

**Fig. 2:**
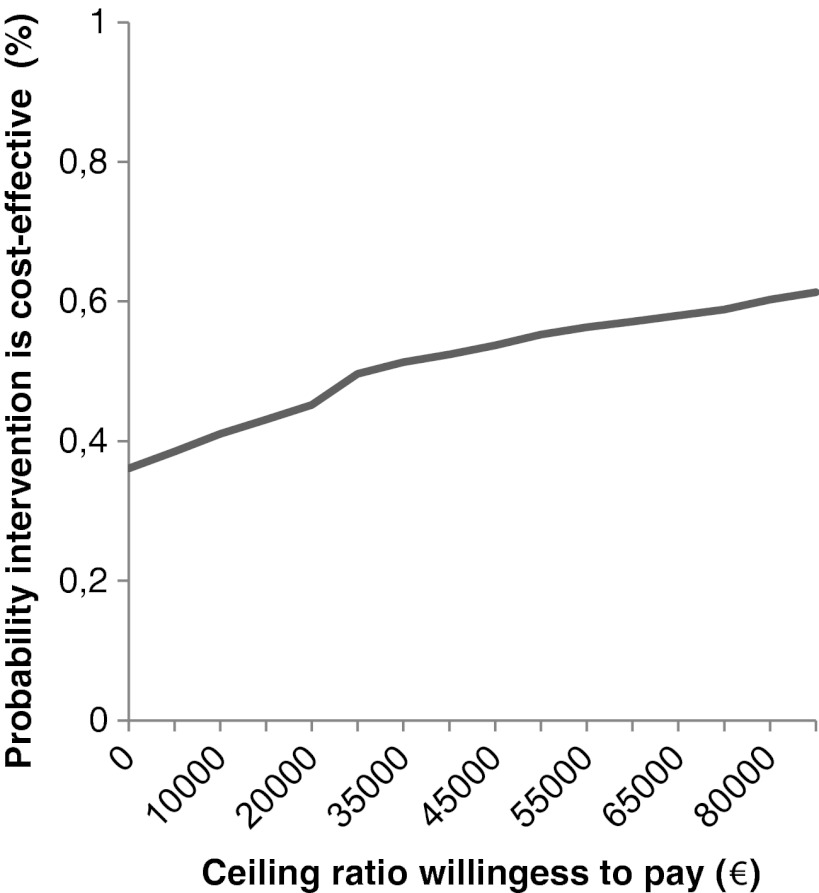
Cost-effectiveness acceptability curve presenting the probability that the nutritional intervention is cost-effective (*y*-axis) for QALY, given various ceiling ratios for willingness to pay (*x*-axis)

At 6 months postoperatively, the intervention effect for QALYs was not statistically significant. The estimate of the intervention effect for change in QALYs was −0.02 (95% CI, −0.12–0.08; *p* > 0.05). The ICER for total societal costs per QALY was 36,943 Euro. As presented in Table 3, the majority of the dots in the CEP based on total societal costs per QALY were located in the NE and SE quadrants. The ICERs located in the NE quadrant represented ratios indicating that the nutritional intervention was more costly and more effective as compared with usual care. The ICERs located in the SE represented ratios indicating that the nutritional intervention was less costly and more effective as compared with usual care. The CEAC (Fig. 2) showed that, with a willingness to pay of 20,000 Euro per QALY, the probability that the nutritional intervention was cost-effective based on its total societal costs per QALY was 45%. If the willingness to pay is 80,000 Euro per QALY, the probability that the intervention is cost-effective increased to 60%.

### Sensitivity analyses

As cost-effectiveness of nutritional intervention may depend on nutritional status and age (co-morbidities and postoperative complications tend to increase with age), sensitivity analyses were performed by stratifying our population for age (55–74 vs. ≥75 years) and nutritional status (malnutrition + risk of malnutrition vs. no malnutrition, according to the MNA). In Table 3, ICERs and the distribution of the ICERs on the CEP are presented for these sensitivity analyses, both for weight and QALYs as outcomes.

**Fig. 3:**
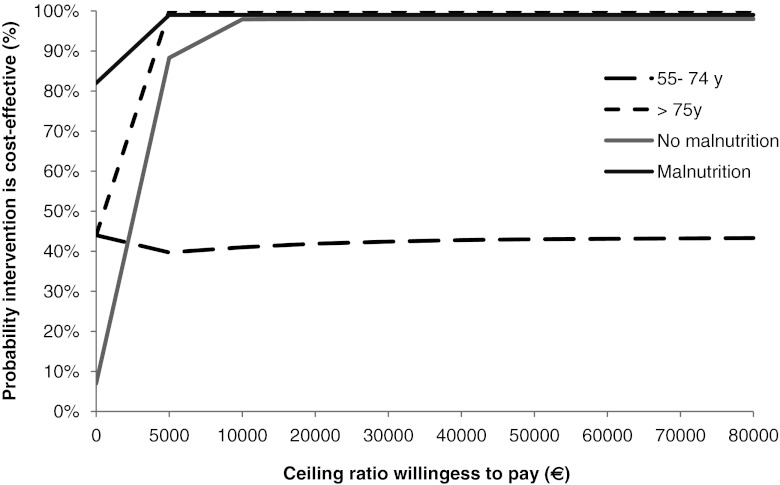
Cost-effectiveness acceptability curve presenting the probability that the nutritional intervention is cost-effective (*y*-axis), given various ceiling ratios for willingness to pay (*x*-axis) with respect to weight increase. Sensitivity analyses performed for age groups and nutritional status at baseline, according to the Mini Nutritional Assessment (MNA)

In Fig. 3, the probability that the nutritional intervention was cost-effective with respect to weight is shown for patients aged 55–74 years and patients aged ≥75 years. In older patients, the probability that the nutritional intervention was cost-effective was 100% if the society would be willing to pay 5,000 Euro or more for 1 kg weight gained. In younger patients, the probability that the intervention was cost-effective was considerably lower (40–44%). As also shown in Fig. 3, in malnourished patients and well-nourished patients, the probability that the nutritional intervention was cost-effective were 100% and 90%, respectively, if the society is willing to pay 5,000 Euro or more for 1 kg weight gain. With a willingness to pay of 2,500 Euro, these percentages would be 90% and ∼50%, respectively, for malnourished and well-nourished patients.

**Fig. 4:**
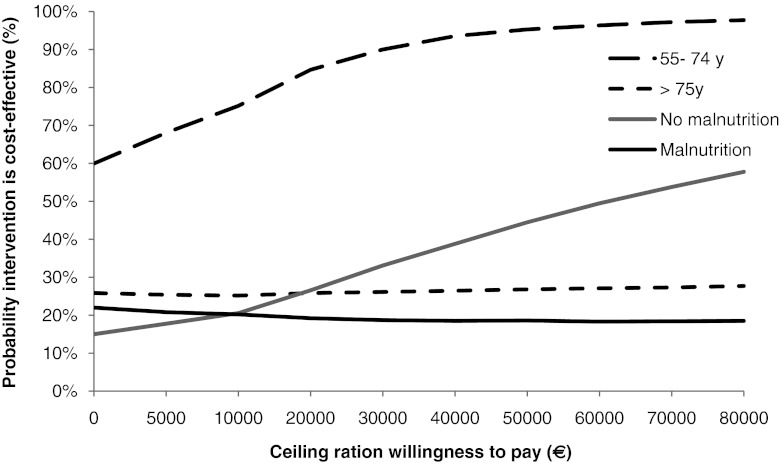
Cost-effectiveness acceptability curve presenting the probability that the nutritional intervention is cost-effective (*y*-axis), given various ceiling ratios for willingness to pay (*x*-axis) with respect to QALY. Sensitivity analyses performed for age groups and nutritional status at baseline, according to the Mini Nutritional Assessment (MNA)

With respect to QALYs, if the nutritional intervention was targeted to patients aged between 55 and 74 years, with a willingness to pay of 20,000 Euro, the probability that the intervention was cost-effective was 85%, compared with only 26% in patients aged 75 years and above (Fig. 4). If the willingness to pay is 80,000 Euro for one QALY, the probability for the nutritional intervention to be cost-effective in the younger group increases to 98% while, in the older group, the probability remains the same. As also shown in Fig. 4, at a willingness to pay 20,000 Euro for one QALY, the probability that the nutritional intervention was cost-effective were 20% in malnourished patients and ∼25% in well-nourished patients. With increasing willingness to pay, the probability that the intervention was cost-effective remained similar in malnourished patients whereas, in well-nourished patients, the probability that intervention was cost-effective increased up to ∼60% at a willingness to pay 80,000 Euro.

## Discussion

Nutritional intervention in elderly hip fracture patients has been proposed as an approach to improve clinical outcome. Despite several decades of research, the overall evidence for the effectiveness of ONS in elderly hip fracture patients with respect to length of stay and functional outcome is limited [42], and no thorough economic evaluation of nutritional intervention in elderly subjects after hip fracture has been performed so far.

In the present study, we assessed the cost-effectiveness of an intensive nutritional intervention combining frequent dietetic counseling and ONS for 3 months postoperatively in elderly hip fracture patients. Results showed that the direct costs of the nutritional intervention were low—613 Euro per treated patient. Total health care costs, patient and family costs, as well as subcategories of these costs were similar in the intervention and control group. Results showed that the nutritional intervention was likely to be cost-effective for weight increase during the intervention period (3 months) in the total study population. Sensitivity analyses with stratification for nutritional status showed that the cost-effectiveness for weight as outcome was especially high in malnourished patients but also (though slightly less high) in well-nourished patients. If the nutritional intervention would be targeted to elderly patients (≥75 years), the probability that the intervention was cost-effective was also high. This was in marked contrast with younger patients (55–74 years), where cost effectiveness was <50%, possibly due to the fact that younger patients generally have a better general condition than elderly patients, so that nutritional intervention will have less effect on their weight.

With respect to QALY, the probability for the intervention to be cost-effective was relatively low for the total population and subgroups; however, the probability that the nutritional intervention was cost-effective with respect to QALY was highest (60–90% depending on willingness to pay) in younger patients (55–74 years).

Our results confirm previous studies indicating that the costs of nutritional intervention are extremely low (in our case, less than 3%) compared with regular health care costs such as hospital costs [20, 22–24, 43, 44]. Previous research in malnourished patients living in the community and in a heterogeneous group of malnourished patients admitted to a mixed medical and surgical ward indicated that nutritional intervention with oral nutritional supplementation alone or combined with dietetic counseling was cost-effective with regard to length of stay [24].

We found that, in hip fracture patients, the probability of the nutritional intervention to be cost-effective with regard to QALY as outcome was relatively low in the older age group of ≥75 years. Of note, older patients more often live in nursing homes even before the fracture, and they tend to have more co-morbidities for which medical treatment is needed; both these factors may overrule the potential cost-reduction induced by the nutritional intervention. Also, after hip fracture, older and malnourished patients may have more postoperative complications and hospital re-admissions as compared with younger and well-nourished patients. As also noted in the literature, medical costs do not seem to be associated with the type of surgical procedure but are mainly determined by increasing age, living in an institution and the presence of co morbidity [21, 38, 41]. Finally, home-dwelling older patients often live alone, which may also result in a higher requirement of professional care as compared with patients living with their partner.

We used a time horizon of 6 months because weight gain or weight maintenance is especially relevant in the vulnerable period after hip fracture, since patients with a poor nutritional status are prone to develop postoperative complications which mostly occur in the first few weeks after hip fracture surgery. In addition, the period of 6 months was chosen because the overwhelming majority of medical costs are made in the first 3 months after hip fracture due to hospitalization, hip fracture surgery, patients’ stay in rehabilitation clinic, their visits to the general practitioner and medical specialist and the treatment of postoperative complications.

It is important to note here that, even though QALYs are often used in cost-effectiveness analyses, this may not be an ideal outcome measure for evaluating effectiveness and cost-effectiveness in elderly patients and in nutritional intervention studies [20, 45]. In the elderly, improvement in nutritional intake and weight may be more clinically relevant, as weight recovery is of vital importance as a basis for overall recovery during the vulnerable period after hip fracture. Also, it may be noted that weight gain is easier to achieve by nutritional intervention than improvement in quality of life, which depends on many other factors than just nutritional status. Moreover, an improvement in weight is necessary to maintain physical activity and cognitive status of the hip fracture patient. In addition, quality of life and resulting QALYs are not only determined by nutrition, but other factors such as loneliness, social support, pain and mobility. Although pain and functional status are included in the EuroQoL 3 level, this questionnaire may not be sufficiently sensitive to detect small differences in quality of life in elderly individuals. Very recently, a new version of the EuroQoL was developed with five response categories. Future research should be performed to detect if the EuroQoL 5 level is sensitive enough to detect small changes in quality of life in the elderly.

Several limitations should be noted. First, although we excluded cognitive impaired patients, volumes of health care consumption might have been influenced by cognitive status of the patients, and therefore these volumes might be over- or underestimated by the patients. Second, the economic analyses were not adjusted for baseline differences between the intervention and control group. Although costs at baseline were similar in both groups, there was a lower proportion of malnourished patients in the intervention group compared with the control group (37% vs. 48%), which might have influenced the overall analyses. However, as cost-effectiveness ratios remained similar in our analyses stratified by malnutrition (yes vs. no), we think this has not influenced our results. Finally, weight at baseline was self-reported because the majority of the hip fracture patients were bedridden at baseline.

We conclude that the additional costs of our nutritional intervention were very low as compared with the total costs. With respect to weight as outcome, the nutritional intervention was likely to be cost-effective. The probability that the nutritional intervention was cost-effective for QALYs was relatively low. Future research should incorporate other outcome measures which are more appropriate for cost-effectiveness evaluations in elderly patients, such as functional limitations, and other outcome parameters relevant for the elderly. Furthermore, effectiveness evaluations should be accompanied with economic and cost-effectiveness evaluations.
